# Soil Communities Promote Temporal Stability and Species Asynchrony in Experimental Grassland Communities

**DOI:** 10.1371/journal.pone.0148015

**Published:** 2016-02-01

**Authors:** Sarah Pellkofer, Marcel G. A. van der Heijden, Bernhard Schmid, Cameron Wagg

**Affiliations:** 1 Department of Evolutionary Biology and Environmental Studies, University of Zürich, Zürich, Switzerland; 2 Plant–Soil Interactions, Agroscope, Institute for Sustainability Sciences, Zürich, Switzerland; 3 Plant–Microbe Interactions, Institute of Environmental Biology, Faculty of Science, Utrecht University, Utrecht, the Netherlands; Chinese Academy of Sciences, CHINA

## Abstract

**Background:**

Over the past two decades many studies have demonstrated that plant species diversity promotes primary productivity and stability in grassland ecosystems. Additionally, soil community characteristics have also been shown to influence the productivity and composition of plant communities, yet little is known about whether soil communities also play a role in stabilizing the productivity of an ecosystem.

**Methodology/Principal Findings:**

Here we use microcosms to assess the effects of the presence of soil communities on plant community dynamics and stability over a one-year time span. Microcosms were filled with sterilized soil and inoculated with either unaltered field soil or field soil sterilized to eliminate the naturally occurring soil biota. Eliminating the naturally occurring soil biota not only resulted in lower plant productivity, and reduced plant species diversity, and evenness, but also destabilized the net aboveground productivity of the plant communities over time, which was largely driven by changes in abundance of the dominant grass *Lolium perenne*. In contrast, the grass and legumes contributed more to net aboveground productivity of the plant communities in microcosms where soil biota had been inoculated. Additionally, the forbs exhibited compensatory dynamics with grasses and legumes, thus lowering temporal variation in productivity in microcosms that received the unaltered soil inocula. Overall, asynchrony among plant species was higher in microcosms where an unaltered soil community had been inoculated, which lead to higher temporal stability in community productivity.

**Conclusions/Significance:**

Our results suggest that soil communities increase plant species asynchrony and stabilize plant community productivity by equalizing the performance among competing plant species through potential antagonistic and facilitative effects on individual plant species.

## Introduction

Understanding the mechanisms behind biodiversity–ecosystem functioning relationships is a major issue in ecology for predicting and maintaining ecosystems in the face of environmental change [1–4]. Previously, it has been shown that higher levels of species diversity, specifically in grassland ecosystems, can maintain ecosystem functioning, and in particular primary productivity [5–9]. Several studies also showed that performance and stability of net aboveground productivity (NAP) of an ecosystem are directly linked to plant community diversity and composition [10–16]. In general, greater stability in ecosystem NAP at higher levels of plant species diversity can be linked to the increased likelihood for species to respond asynchronously to environmental perturbations, thus stabilizing the overall performance of the community through time [17,18]. This can be associated with the increased probability of niche differentiation that occurs among the species at higher diversity levels [14,19–21].

Considering the importance of plant species diversity in stabilizing NAP during environmental perturbations, it is critical to consider ecological mechanisms that support plant community diversity and mediate their temporal performance. For instance, soil communities are well known to influence multiple ecosystem functions [22–25], with particular effects on plant competition and the overall performance and composition of a plant community [22–27]. Considering that diversity and composition of the soil community have a strong influence on the performance of individual plant species and plant community composition, it is likely that the interaction of plants with soil communities may be an underlying mechanism influencing the stability of plant community productivity. Thus, soil organisms that alter the performance of individual plant species within a community could potentially increase or decrease the stability of plant community productivity by altering temporal competition dynamics among the plant species as the plant community develops and responds to environmental variation [28]. This is of critical importance since it is now known that many anthropogenically managed ecosystems show altered soil community composition as well as the suppression and loss of key groups of soil organisms that can alter the plant community performance and composition [29–33]. Only recently, there has been some evidence to suggest that the suppression of key soil biota, such as mycorrhizal fungi, may be linked with stability in NAP [34]. However, there is currently little evidence to know whether soil communities overall influence plant community stability.

Here we investigate the importance of the soil community for supporting temporal stability in the NAP of a grassland plant community and the temporal asynchrony among plant species as the plant community develops. Considering the connections previously found between the presence of soil biota and plant community performance, we hypothesize that the soil communities with which the plant community interacts will not only support a high diversity and NAP in the plant community, but will also promote plant species asynchrony and the stability in the community productivity. To address our hypothesis, we established a grassland plant community in a standardized sterile soil substrate inoculated with either a natural unaltered soil community, or the same inoculum, but sterilized to remove the natural soil biota.

## Materials and Methods

### Soils and inocula

Experimental microcosms were set up using 42 three-liter pots (19 cm diameter x 14.5 cm height) that were sterilized by autoclaving. Each pot was filled with 2.25 kg (dry mass) substrate of a 50/50 field soil/quartz sand mix that was sieved through a 5 mm mesh and sterilized by autoclaving (120°C for 90 minutes). The field soil used as the sterile substrate in each microcosm came from a natural grassland near the Agroscope Reckenholz research station in Zürich, Switzerland (47° 25’ 38.71” N, 8° 31’ 3.91” E). The sterilized field soil was inoculated with 125 g of one of the six possible inocula treatments: soil inoculum from three sites with different management practices × two soil community treatments—unaltered or sterilized. The inocula were mixed throughout the substrate prior to planting. Each of the six soil inocula treatments was replicated seven times for a total of 42 experimental communities.

The soil inocula were collected from three agricultural fields with different management histories. We used soils from these different management practices to better generalize our results independent of site-specific histories and characteristics. All sites from where our study's soil samples were collected did not host endangered or protected species. With the permission of Jochen Mayer of Agroscope and Paul Mäder of the Institute of Organic Agriculture (FiBL), we were allowed to collect two of the soils from FiBL’s so-called DOK experimental field site in Therwil, Switzerland (47° 30' 8.9964” N, 7° 32' 21.8292” E). This experiment was designed to assess different agricultural management practices, such as conventional and organic management, on various ecological and agricultural characteristics of plots (see [29] for details). For the present study soil was collected from four plots where the management practice was the addition of organic fertilizer (Site A, organic) and from another four plots where the management practice was addition of mineral fertilizer (Site B, conventional). The third soil was sampled, with the permission of the landowner, from their privately owned agricultural plot in Freiburg, Germany (47° 58' 26.058” N, 7° 46' 31.5336” E). This site had been continuously planted with the same crop species (maize) for more than 10 years (Site C, intensive). Details about soil characteristics of the different soil treatments are provided in S1 Appendix in Supporting Information.

At all three sites soil was collected using four transects, one meter apart per plot, coring soil every four meters. Soil cores were mixed per site and homogenized by sieving through a 5 mm sieve. Half of the soil from the three sites was sterilized by autoclaving (120°C for 20 min). This resulted in two inocula treatments per site; a sterile soil inoculum and an unaltered soil inoculum (*sensu* 27,28,35). Autoclaving soil is well known to eliminate the presence of mycorrhizal fungi and severely reduce the microbial community [23,35–37]. The inocula volume only made up approximately 5% of the total soil volume to minimize the possible abiotic effects of inocula sterilization in our model systems. We used root colonization by arbuscular mycorrhizal fungi (AMF) at the end of the experiment (55 weeks post initial inoculation) as an indicator as to whether differences between our unaltered and sterilized soil inoculation treatments remained after 1 year. Although AMF colonization is only one component of soil community composition, the absence or presence of AMF is an effective indicator that a key component of the soil microbiota have been effectively eliminated or severely suppressed. Ultimately, AMF colonization was very different between the two soil inocula treatments (F_1, 37_ = 122, P < 0.0001). AMF colonization in the sterile treatment was on average 5.67% and was not statistically different from zero (95% confidence interval = -0.23 to 11.1). Conversely the unaltered soil inoculum treatment had a mean colonization of 60.4% (95% confidence interval = 54.9, 65.8), indicating the sterilized soil inoculum treatment resulted in a suppressed soil biotic community throughout the experiment.

### Plant community

In Fall of 2012 each microcosm was planted with six individuals of the grass *Lolium perenne* and six individuals of the nitrogen-fixing legume *Trifolium pratense*, along with one individual of *Achillea millefolium* (forb), *Festuca pratensis* (grass), *Lotus corniculatus* (legume), *Plantago lanceolata* (forb), and *Prunella vulgaris* (forb), for a total of 17 individual plants per pot. These plant species commonly co-occur in European grasslands [38]. Moreover, this specific mixture made up largely of *T*. *pratense* and *L*. *perenne*, was selected because the two main species commonly co-occur and are extensively used in land management as crop in fallow years on agricultural fields or establishment as fodder crops. Additionally, *T*. *pratense* and *L*. *perenne* are model species for studying temporal dynamics in plant communities due to their complementary use of the biotope that results in their overyielding [39,40]. Moreover, legumes depend heavily on associations with their soil biota for increased performance [23,26,41]. We included the five other plant species in the experimental communities at a lower abundance because they commonly occur in managed grass-clover fields, and they also allow for a better assessment of plant community compositional responses (e.g. diversity, evenness).

Seeds of each species were surface sterilized by immersion in 2.5% hyposodium chlorate for five minutes, then rinsing thoroughly in distilled H_2_O. Surface-sterilized seeds were then plated onto 1% Agar in Petri dishes to germinate. In order to ensure that the seeds of all species were at the same stage of development when planted, the seed germination process was staggered so that each species exhibited the presence of cotyledon(s) and/or radicle when transplanted. Seedlings were planted into one of 17 evenly spaced and randomly selected positions in the inoculated substrate of each microcosm. These experimental communities were set up over two days. In subsequent analysis of variance (ANOVA), the set up day was used as a blocking factor.

These experimental communities were established in a glasshouse compartment where natural light was subsidized by 400-W high-pressure sodium lamps in order to maintain an environment of 16 h / 25°C days and 8 h / 16°C nights with a light level above 300 W/m2. Twice weekly, the microcosms were watered to maintain gravimetric soil moisture in the range of 10–20%. However, since the greenhouse conditions maintain a constant environment, which does not reflect those found in nature, which might allow for variation in plant species competitive interactions through time, we induced a variation in the watering regime to simulate environmental variation in precipitation. The variation in precipitation was applied to all of the experimental communities at the same time by withholding watering for 10 days beginning five and a half weeks before each harvest. The plant communities were grown under these conditions for a total of 55 weeks (~1 year), with five harvests starting 11 weeks after planting and occurring every 11 weeks after that.

### Data collection

Over the 55-week growing period plant individuals were cut at 5 cm above the soil surface every 11weeks. Plants were harvested from the experimental communities according to the same schedule in which they were planted. Plant individuals were counted and separated by species, dried at 65°C and the biomass weighed. For each harvest we calculated the net aboveground productivity (NAP), and three measures of plant community diversity, as suggested in work by Jost [42,43]. These diversity measures included plant realized species richness, Shannon diversity (H’), and inverse Simpson diversity 1/D = $\sum {\left(\frac{p_{i}}{p_{t}}\right)}^{2}$. We included these three measures of true diversity as they all utilize differing degrees of abundance to assess the diversity of a community. Community evenness was calculated as: $E_{var}\;=1-2/\pi \times \text{arctan}\;\{\sum_{i=1}^{S}\;{\left(\text{ln}(p_{i}\;)-\sum_{t=1}^{S}\frac{\text{ln}(p_{t})}{S}\right)}^{2}\;/S\}$, as proposed by Smith and Wilson[44]. In these equations *S* is the number of species in the sample and *p*_*i*_ is the abundance of the *i*-th species and *p*_*t*_ is the total community biomass.

Plant species asynchrony was calculated for each experimental community as 1 − *φ*_*b*_, where *φ*_*b*_ is species synchrony, calculated by $\varphi_{b}=\frac{\sigma^{2}}{{\left(\sum_{i-1}^{S}\sigma_{i}\right)}^{2}}$, where σ^2^ is the variance in NAP over time and σ_i_ is the temporal standard deviation of the *i*-th species in each experimental community as defined by Loreau & de Mazancourt [19]. Since our experimental design utilized a plant community dominated by a common grass-clover mixture, we also assessed the asynchrony among plant functional groups using the above-mentioned equation for asynchrony with σ^2^ being the variance in the sum of the biomass of two plant functional groups and σ_i_ as the temporal standard deviation of plant functional group *i*. Considering this additional level of community grouping, beyond the individual species, has been shown to be of particular importance for capturing a more accurate picture of the effects of diversity on ecosystem stability [45] and how that stability scales when moving up the hierarchy of organizational levels [46]. We calculated temporal stability in both NAP of the whole community and of each individual plant species using the inverse coefficient of variation determined by μ/σ, where μ is the overall temporal mean of each community or species’ NAP and σ is the standard deviation of NAP over time [18,19,47].

### Data analysis

All data analysis and statistics were completed using R software (version 3.0.0) and in all analysis significance was determined as a type I error of α < 5%. The R package ‘vegan’ was used to calculate diversity indices. Plant community characteristics that were repeatedly measured throughout the experiment (NAP, richness, Shannon diversity, inverse Simpson diversity and evenness (E_var_) were analyzed using the package ‘ASReml’ for R (VSN International) in order to include the autoregressive structure to account for temporal correlation in the mixed effects model. In the mixed effects models, all above-mentioned community characteristics were assessed for the differences between the two soil inoculum treatments and the interaction with the harvest time point as fixed effects. The experimental block and identity of the microcosm were added as random intercepts. Since we were specifically interested in the general effects of the soil community on the temporal performance of the plants in a community context, the management history was also included as a random effect and its interaction with the soil inoculation treatment as random intercepts (but see S2–S5 Appendixs for site-specific effects).

The temporal stability in the NAP, the temporal standard deviation of NAP and the performance of individual plant species, as well as the temporal asynchrony among plant functional groups were assessed for differences between unaltered and sterilized soil community treatments with only the soil community treatment as a fixed effect in the model using ‘lme4’, and ‘lmerTest’ packages in R for mixed effects analysis of variances [48]. The temporal standard deviation of NAP was analyzed to assess how differences in stability (μ/σ) between soil treatments were affected by differences in the mean (μ) and the temporal variation (σ) separately [49,50].

## Results

**Fig 1:**
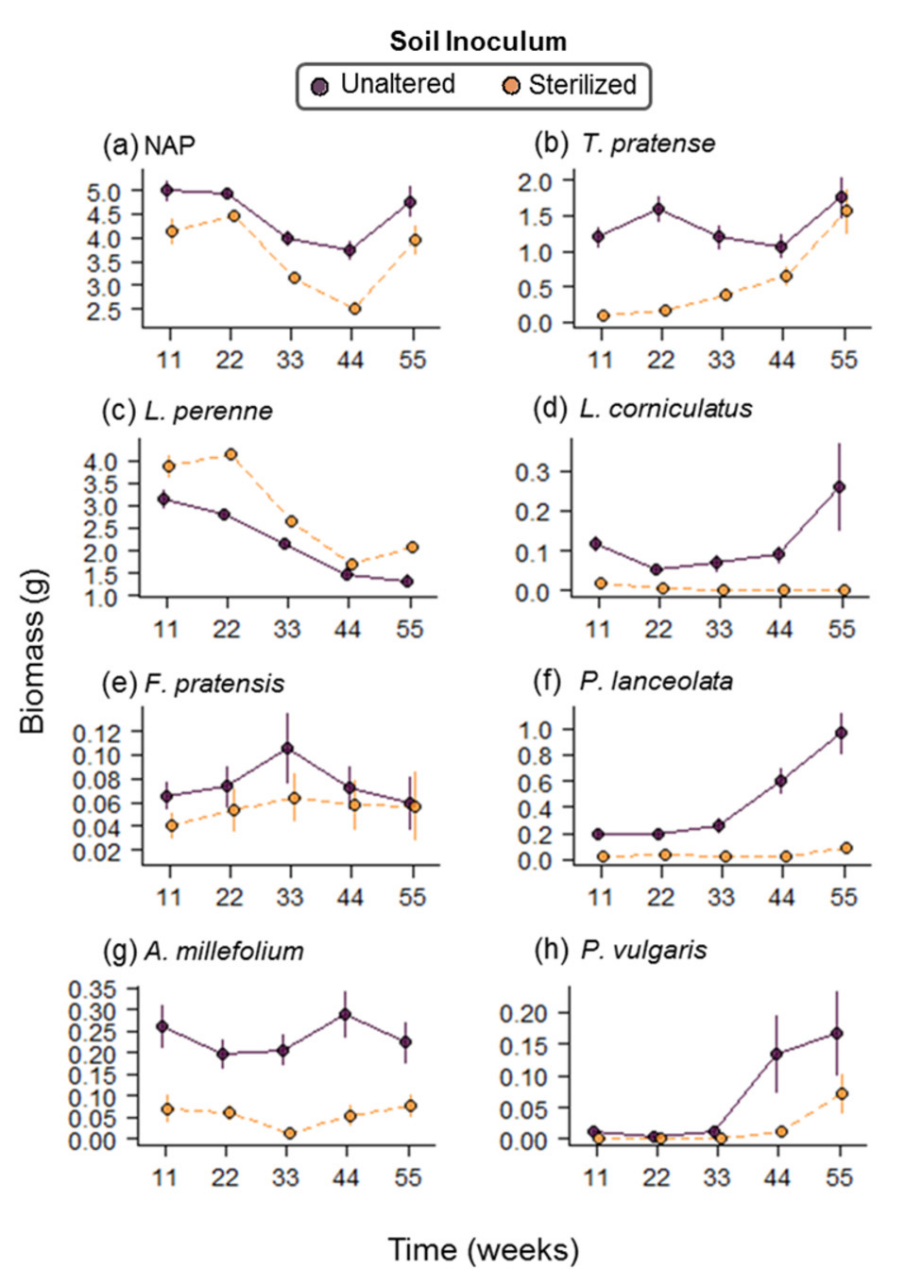
Net and species specific productivity when inoculated with the sterilized and unaltered soil inoculum. Means with standard errors of the mean are shown for (a) NAP and (b–h) the individual plant species at each harvest when grown with sterilized soil inoculum (light points, dashed line) or unaltered soil inoculum (dark points, solid line). Lines connecting means highlight the trend between consecutive harvest time points.

**Fig 2:**
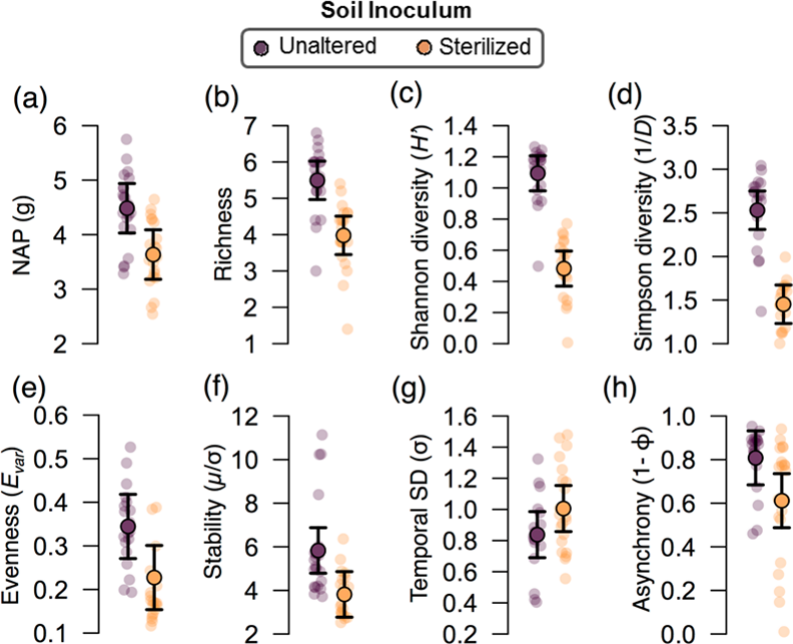
Temporal mean of plant community characteristics when inoculated with the sterilized and unaltered soil inoculum. Means with 95% confidence intervals for the pair-wise difference between the unaltered and sterilized soil community treatments are shown for (a) NAP, (b) realized plant species richness, (c) Shannon diversity, (d) inverse Simpson diversity, (e) evenness, (f) temporal stability in NAP, (g) asynchrony among plant species, and (h) the temporal standard deviation of NAP of plant communities when inoculated with unaltered soil or (dark points) or sterilized (light points) soil. Lightly shaded individual points are values for individual microcosms.

The sterilized soil inoculum resulted in lower net productivity in the plant communities (Table 1, Fig 1A & Fig 2A). The net performance of the communities (NAP) and their variation overtime is a consequence of the response of the individual plant species to the soil inocula treatments. Here all the plants species were less productive with the sterilized soil inocula with the exception of the predominant grass *L*. *perenne* (Fig 1B–1G). Consequently, plant species richness, Shannon diversity, inverse Simpson diversity, and evenness were reduced by the inoculation with a sterile soil inoculum (Table 1, Fig 2B–2E). All plant community characteristics were also found to vary through time (Table 1, S6 Appendix). The unaltered soil inoculum resulted in a more stable NAP through time than the community inoculated with sterilized soil (Fig 2F). Moreover, the sterilized soil inoculum also resulted in a higher temporal standard deviation in NAP (F_1, 35_ = 4.94, P = 0.033; Fig 2G) indicating that the decline in stability of NAP through time (μ/σ) with sterilized soil inoculum resulted from an increase in the temporal variation in the NAP (σ shown in Fig 2G) as well as a decline in the overall temporal mean in the NAP (μ shown in Fig 2A). Asynchrony among individual plant species was also found to decline when plant communities were inoculated with sterile soil (F_1, 36_ = 9.90, P = 0.003, Fig 2H).

**Table 1:** Summary of ANOVA results for the effects of soil inoculum treatment on plant community characteristics.

NAP	DF_num_	DF_den_	F	
Harvest (H)	4	115	45.27	***
Inoculum (I)	1	37.7	13.72	***
H × I	4	115	2.04	*
**Richness**				
Harvest (H)	4	123.9	11.52	***
Inoculum (I)	1	37.0	31.57	***
H × I	4	123.9	1.45	
**Shannon diversity (H’)**				
Harvest (H)	4	157.0	11.18	***
Inoculum (I)	1	47.0	9.97	**
H × I	4	157.0	1.87	
**Inverse Simpson diversity (1/D)**				
Harvest (H)	4	145.9	31.55	***
Inoculum (I)	1	31.3	115.90	***
H × I	4	145.9	3.59	**
**Evenness (Evar)**				
Harvest (H)	4	147.1	26.84	***
Inoculum (I)	1	35.7	91.51	***
H × I	4	147.1	2.22	*

Inoculum refers to the inoculum treatment (unaltered versus sterilized) and harvest to the harvest period (at 11, 22, 33, 44 and 55 weeks). The response variables are the net aboveground productivity (NAP), realized richness, Shannon diversity, inverse Simpson diversity, and evenness.

* = P < 0.1

** = P < 0.01

*** = P < 0.001

DF_num_ = numerator degrees of freedom, DF_den_ = Kenward-Roger adjusted denominator degrees of freedom, F = variance-ratio.

**Fig 3:**
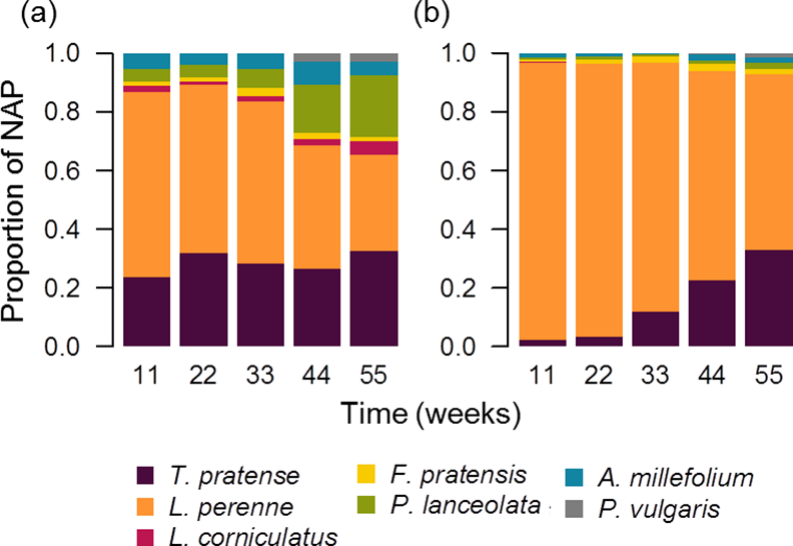
Plant species composition at each harvest with two soil inocula treatments. Plant community composition as represented by the species proportional abundance is shown for (a) the unaltered soil inoculum and (b) the sterilized soil inoculum. Colored bar height indicates the proportion of each plant species.

**Fig 4:**
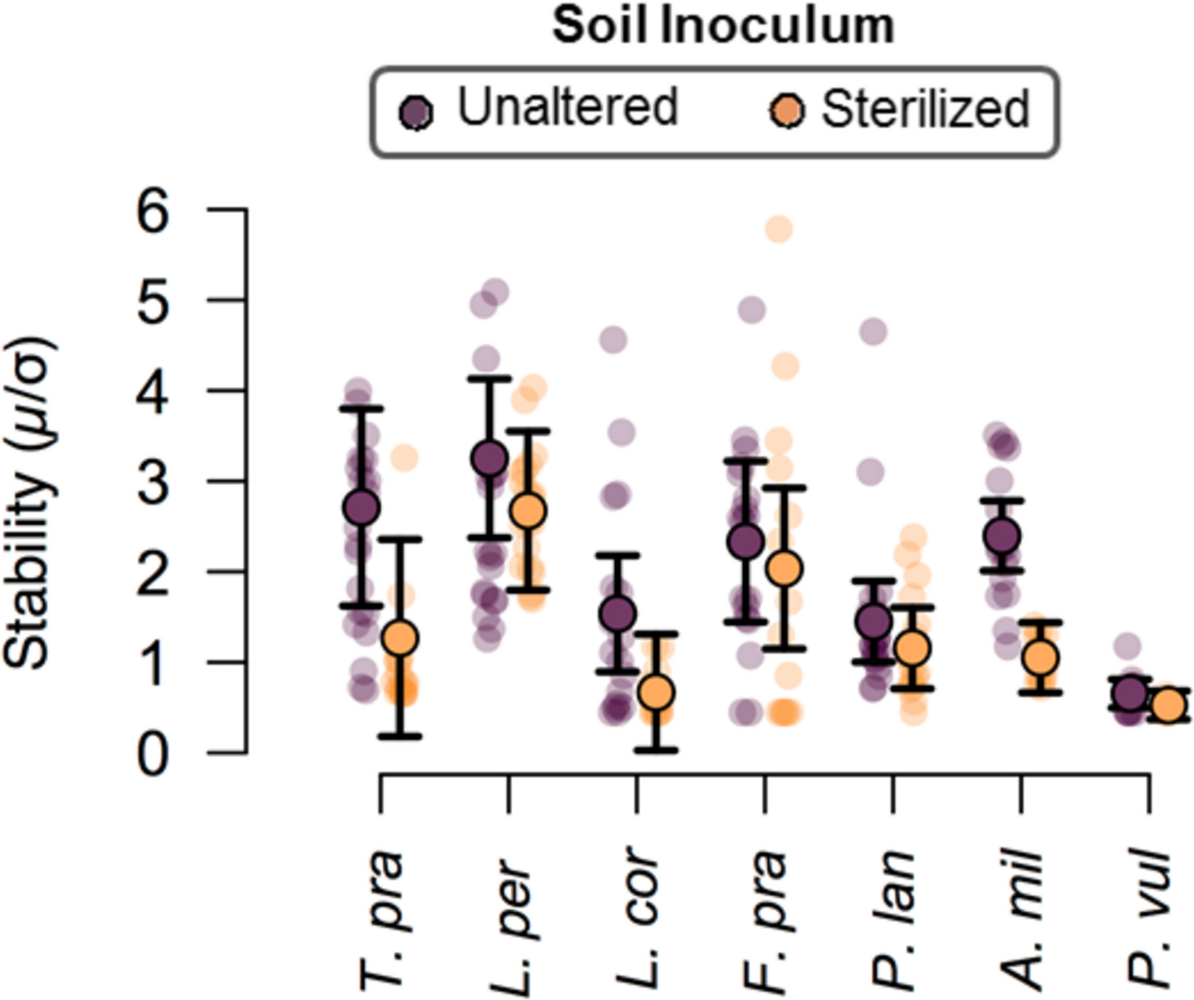
Temporal stability of individual plant species and their contribution to the temporal variation in NAP. Mean values with 95% confidence intervals are shown for the stability of individual plant species. Plant communities inoculated with the unaltered soil are indicated by dark points and light points indicate plant communities inoculated with the sterilized soil. Lightly shaded individual points are values for individual microcosms.

**Fig 5:**
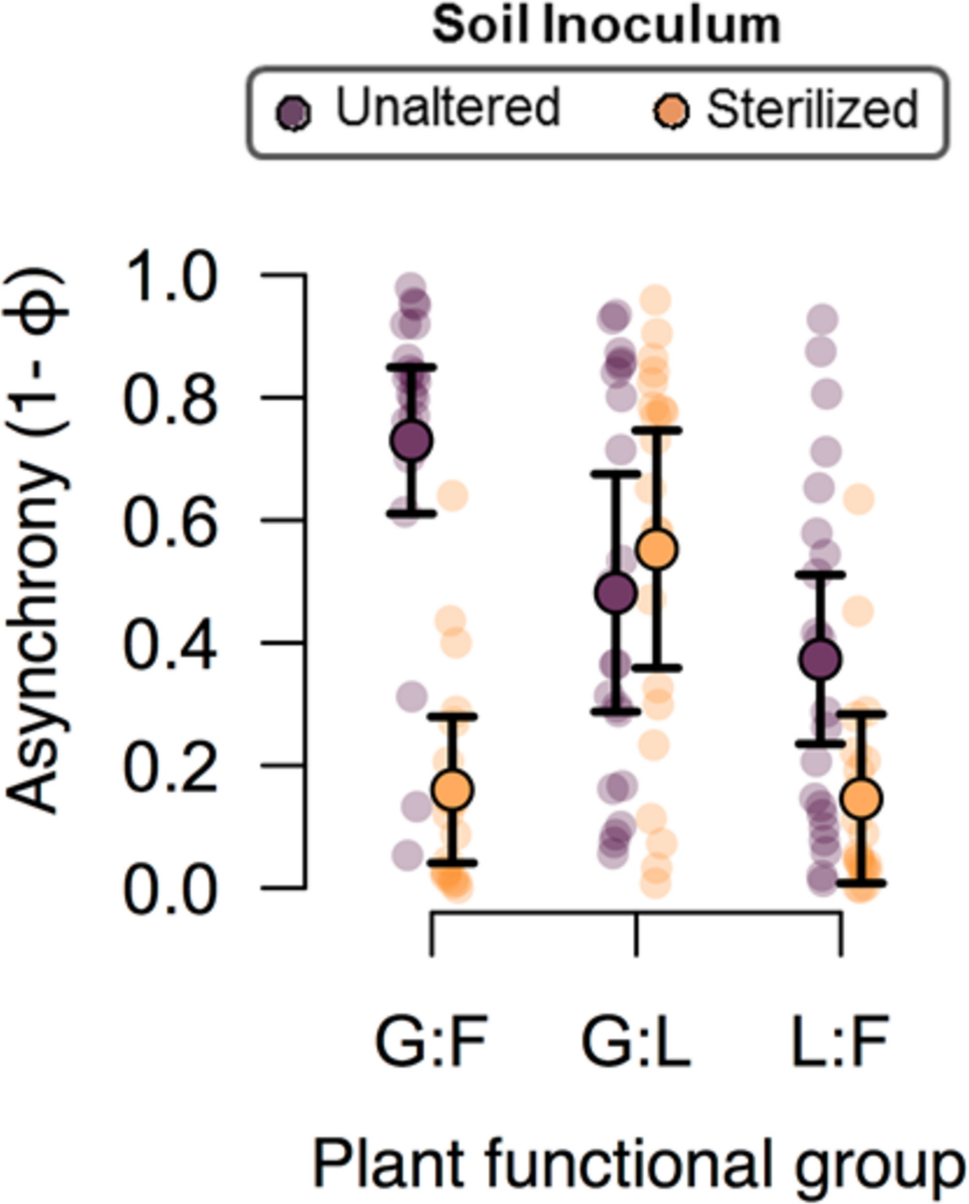
Mean asynchrony between pairs of plant functional groups in the two inoculum treatments. The unaltered soil inoculum is shown in darkly colored points and the sterilized inoculum treatment in light colored points. Bars represent 95% confidence intervals. G = grasses, F = forbs, L = legumes. Lightly shaded individual points are values for individual microcosms.

The difference in the effect of the two soil inoculum treatments on the plant composition was markedly observed in the proportional abundance of each species between the two soil inoculum treatments, where the species were much more proportionally represented when associated with the unaltered soil inoculum (Fig 3A). Conversely, *L*. *perenne* was much more predominant in the plant community when inoculated with the sterilized soil, where all other species combined contributed to less than 50% of the overall community productivity (Fig 3B). Additionally, individual plant species were also generally less variable over time when inoculated with the unaltered soil inoculum (Table 2, Fig 4). Specifically, the stability in the performance of *A*. *millefolium* and the legumes *L*. *corniculatus* and *T*. *pratense* were most negatively affected by the sterilization of the soil inoculum (Fig 4). Furthermore, the effect of the soil community treatment on the asynchrony between different functional groups depended on the functional group pairing (Fig 5, F_2,80_ = 16.2, P<0.0001). Specifically, the unaltered soil community promoted asynchrony between grasses and forbs and between forbs and legumes but not between grasses and legumes (Fig 5).

**Table 2:** ANOVA results for effects of soil inoculum on biomass stability.

		Stability	
	DF_num_	DF_den_	F
*T*. *pratense*	1	36.0	6.81*
*L*. *perenne*	1	37.0	1.67
*L*. *corniculatus*	1	29.1	5.77*
*F*. *pratensis*	1	29.7	0.42
*P*. *lanceolata*	1	32.2	1.65
*A*. *millefolium*	1	29.0	41.0***
*P*. *vulgaris*	1	16.8	1.99

* = P < 0.05

*** = P < 0.001

DF_num_ = numerator degrees of freedom, DF_den_ = Kenward-Roger adjusted denominator degrees of freedom, F = F-ratio.

## Discussion

It has been well documented that plant community composition is altered by various soil biota, such as pathogens, decomposers and symbiotic fungi [22,23,27,28,33,51,52]. Paralleling these past studies, we found that the unaltered soil community maintained higher plant species diversity (richness, Shannon diversity, and 1/Simpson diversity) and resulted in a more even plant community with greater NAP than plant communities with a sterilized soil community. More importantly, in line with our hypotheses, we found that the unaltered soil communities led to higher stability in community NAP and maintained a higher asynchrony among plant species than the sterilized soil community as our experimental plant communities developed over the course of the experiment. The higher stability in NAP resulted from a combination of both a higher temporal mean NAP and a lower temporal standard deviation in plant communities that were associated with the unaltered soil communities.

Greater stability in more species-rich grassland communities is often observed to be associated with lower stability in the performance of individual plant species due to strong asynchronous fluctuations among plant species that result from combinations of environmental, demographic and competitive fluctuations [18,19,53,54]. However, unlike previous studies, we found greater stability in the performance of individual plant species in communities where the overall NAP was more stable. Specifically, the unaltered soil inoculum in our study resulted in greater performance and stability of individual plant species such that more species were able to contribute to the NAP and its variation through time. Conversely, with the sterilized soil community, the NAP of the plant communities was largely driven by the dominance of the grass *L*. *perenne*, such that the temporal variation in the subdominant species had little effect in stabilizing NAP across time. This indicates that the variation in the productivity of *L*. *perenne* could not be sufficiently compensated for by the productivity of the other species when the soil community was sterilized, which is also indicated by the lower species asynchrony with the sterilized soil community. This corresponds with previous findings that a higher evenness in the performance of plant species, often as a result of greater plant species richness, is a key component behind the stability in the NAP of a community and may be suggestive of greater species asynchrony [55–58].

Greater stability in individual plant species has been shown to occur when species have certain growth-stabilizing functional traits, like shallower rooting depth, denser leaves, or reduced growth when water becomes more available [59]. Here the greater stability in the individual species and the asynchrony among plant species, as well as between plant functional groups, likely reflects species resource acquisition traits, such as the dependence of forbs and legumes on soil mutualistic microbes to acquire and compete for soil resources. For instance, the influence that soil biota can have on the performance of plants has been well known to shift plant–plant competitive interactions between grasses, legumes and forbs that ultimately shapes plant community composition [27,28,33,60–64]. The greater diversity and evenness in our plant communities could have been a direct effect of soil mutualists, such as rhizobia in legumes and mycorrhizal fungi in both forbs and legumes. These soil mutualists aid forbs and legumes to acquire soil resources, thus improving the plants’ aboveground productivity as well as their ability to compete with neighboring plants, such as grasses [41,60,62,64–66]. Thus, the dependence of some plants on soil mutualistic microbes in our study, such as *T*. *pratense*, likely resulted in soil resource limitation that resulted in the small and slow increase in productivity over time. Similarly, Yang *et al*. [34] also reported that the suppression of mycorrhizal fungi altered the dominance of particular plant species, and reduced the performance of N-fixing forbs that inhibited the overall temporal stability in the performance of a grassland ecosystem. The sharp difference in the effect of the soil community on the asynchrony between the grasses and forbs in our study may indicate that the soil biotic community mediates the temporal soil resource acquisition between these plant functional groups.

The presence of plant species-specific soil pathogens could have also reduced the temporal performance of the grasses, such as *L*. *perenne*, when inoculated with the unaltered soil. For example, van der Putten & Peters [28] observed that competition between two grasses over a 16-week period was altered by the sterilization of rhizosphere soil biota, and that the competitive suppression of the subdominant plant over time was increased by sterilization of rhizosphere biota. Considering these studies, it would seem that the temporal variations in the performance of individual plants species can be driven in part by the soil community, through direct and indirect beneficial and antagonistic plant-soil community associations, which may alter competitive dynamics among plant species as the community develops. However, further work is need to directly assess how soil mutualisms and pathogens affect plant-plant competitive dynamics and temporal resource partitioning that drive the overall plant community asynchrony and stability.

Our results indicate that the complexity of the belowground soil community with which plants interact can influence the temporal performance of individual plant species and potentially the competitive interactions among plants. This leads to greater species asynchrony and the overall stability in the performance of the plant community [67]. However, as some of the plant communities grew to a size where the pots potentially limited their growth (> 1g biomass per liter of soil substrate) [68], further investigation as to the role soil communities play in shaping the temporal dynamics in plant communities under natural field conditions are need. Furthering such findings in the future may be of key importance for land management practices where the diversity and the presence of various groups of soil biota are frequently found to be suppressed by increased anthropogenic activity [29–32]. However, additional efforts are needed to better elucidate the more finite mechanisms by which the various components of the soil community (i.e. pathogens or mutualisms) drive asynchrony among plant species and stabilize ecosystem NAP in both managed and unmanaged ecosystems.

## Supporting Information

S1 Appendix Inocula soil history with initial soil properties analyses results. (TIF)

S2 Appendix ANOVA results for responses in plant community characteristics to inoculum site origin, treatment and harvest. Plant community characteristics are net aboveground productivity (NAP), richness, Shannon diversity (H’), inverse Simpson diversity (1/D), and evenness (Evar). Density (the total number of individual plants in each community), harvest period, the soil inocula treatment, the site (source of the soil inoculum) and all interactions were considered as fixed effects. Model random effect terms are also provided.
(TIF)

S3 Appendix ANOVA results for the response in stability and species asynchrony to the soil inocula treatments and the inoculum site origin. (TIF)

S4 Appendix Figure showing plant community characteristics in relation to the inoculum site origin. Mean values with 95% confidence intervals are provided for the (a) NAP, (b) richness, (c) Shannon diversity, (d) inverse Simpson diversity, (e) evenness, (f) community stability, and (g) species asynchrony of plant communities with an unaltered soil community (dark points) and sterilized soil community (light points) for the three sites averaged over the full duration of the experiment.
(TIF)

S5 Appendix ANOVA results for the effect of the soil community treatments on the stability in the biomass of individual species and the covariance between the individual plant species and NAP. (TIF)

S6 Appendix Figure showing plant community characteristics in relation to the inoculum treatments at each harvest point. Mean values are shown with 95% confidence intervals of plant (a) NAP, (b) richness, (c) Shannon diversity, (d) inverse Simpson diversity, and (e) evenness for each harvest the unaltered (dark points) and sterilized (light points) soil community treatments.
(TIF)
