# Supplementary figures and images for: Soil Communities Promote Temporal Stability and Species Asynchrony in Experimental Grassland Communities
Source: PLoS One. 2016 Feb 1;11(2):e0148015. doi: 10.1371/journal.pone.0148015 (PMC4734741; doi:10.1371/journal.pone.0148015)

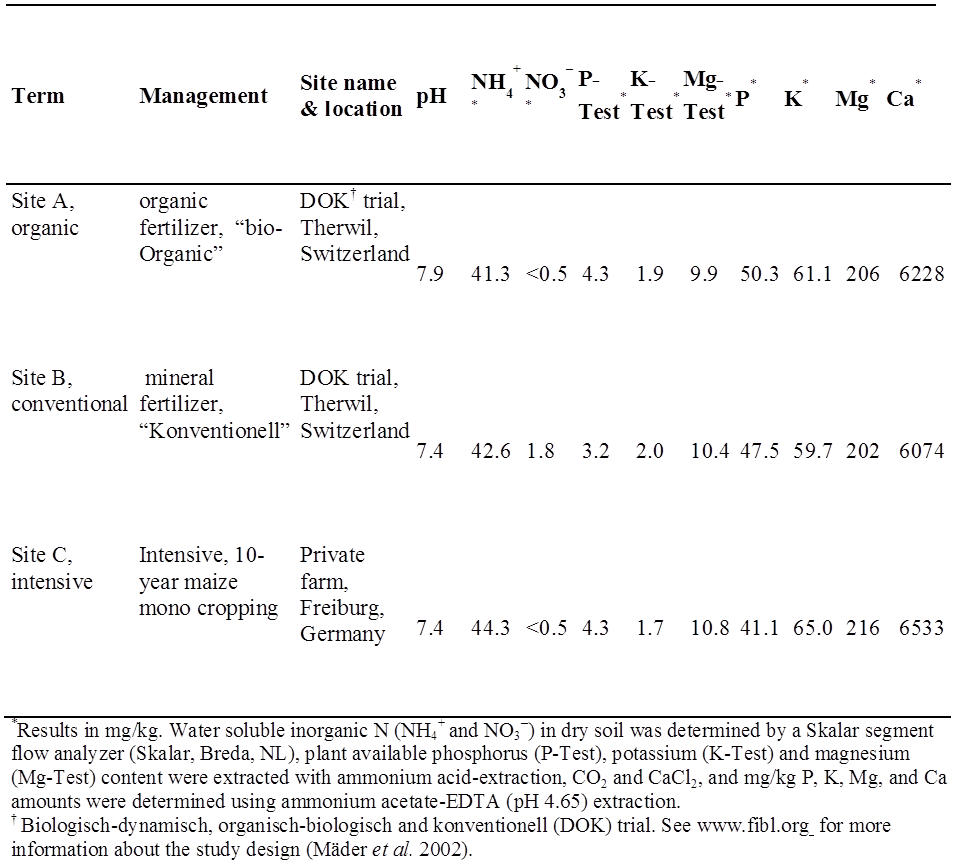

Supplement: S1 Appendix — (TIF) [file pone.0148015.s001.tif]

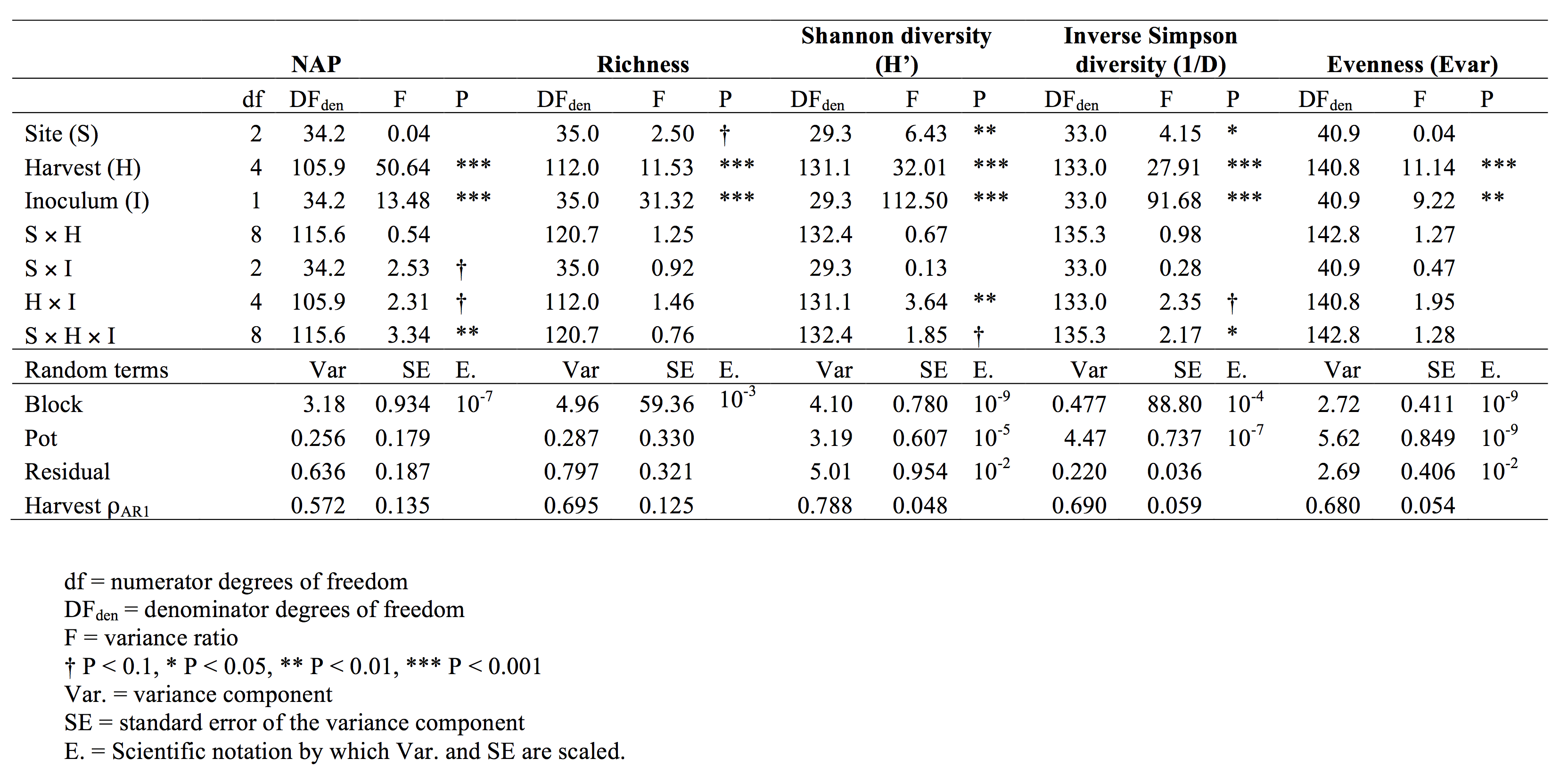

Supplement: S2 Appendix — Plant community characteristics are net aboveground productivity (NAP), richness, Shannon diversity (H’), inverse Simpson diversity (1/D), and evenness (Evar). Density (the total number of individual plants in each community), harvest period, the soil inocula treatment, the site (source of the soil inoculum) and all interactions were considered as fixed effects. Model random effect terms are also provided. (TIF) [file pone.0148015.s002.tif]

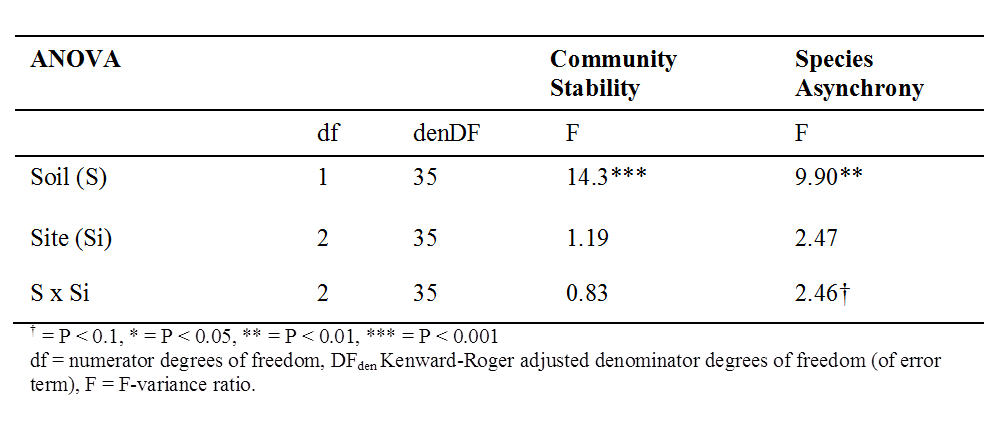

Supplement: S3 Appendix — (TIF) [file pone.0148015.s003.tif]

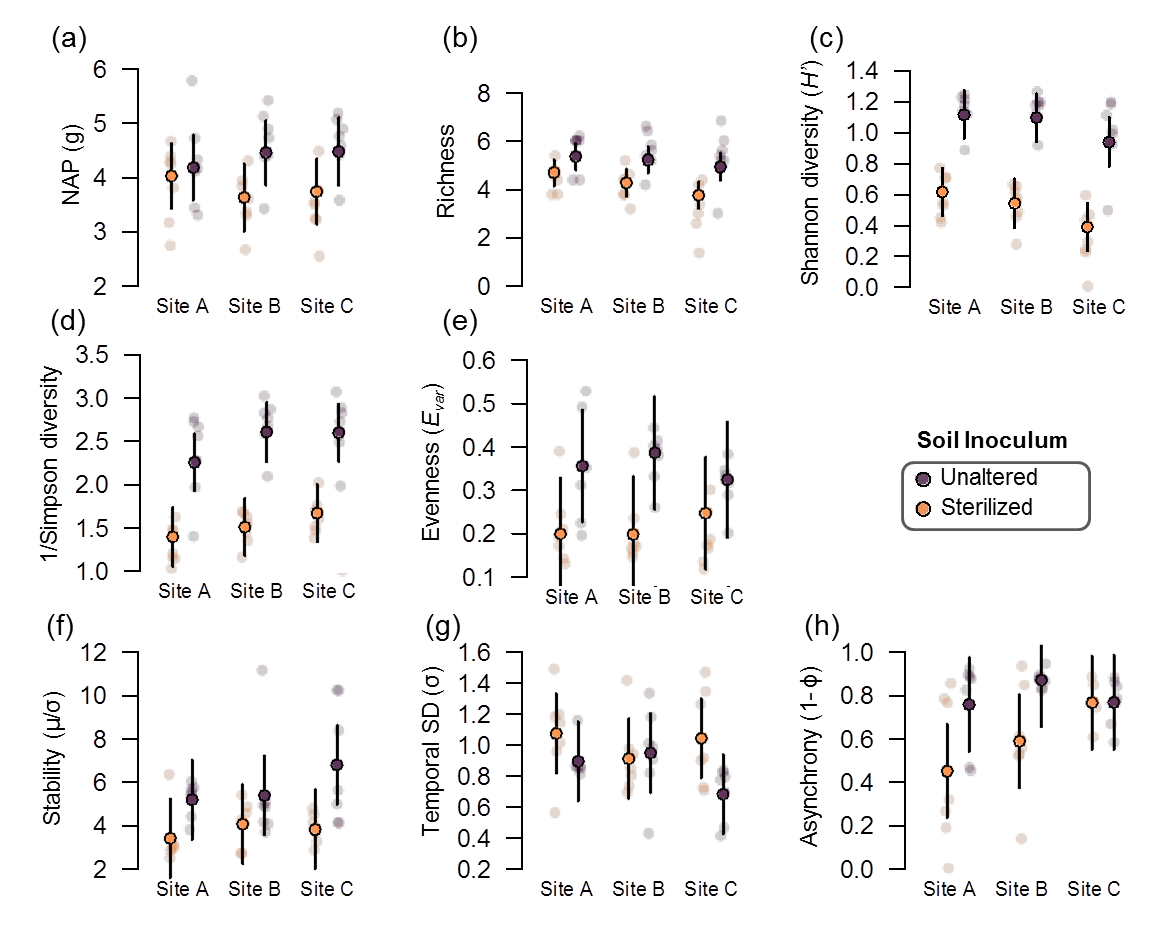

Supplement: S4 Appendix — Mean values with 95% confidence intervals are provided for the (a) NAP, (b) richness, (c) Shannon diversity, (d) inverse Simpson diversity, (e) evenness, (f) community stability, and (g) species asynchrony of plant communities with an unaltered soil community (dark points) and sterilized soil community (light points) for the three sites averaged over the full duration of the experiment. (TIF) [file pone.0148015.s004.tif]

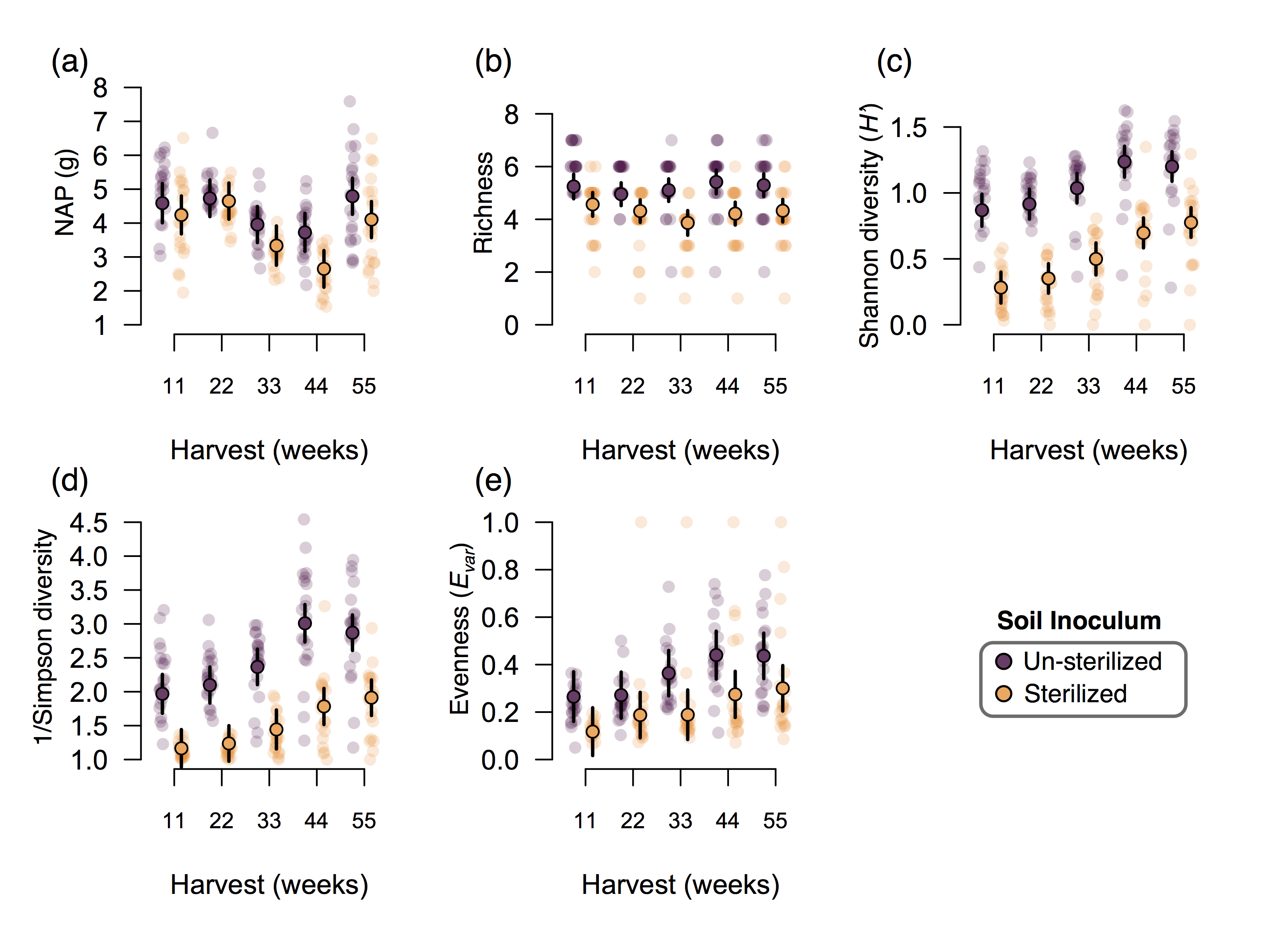

Supplement: S6 Appendix — Mean values are shown with 95% confidence intervals of plant (a) NAP, (b) richness, (c) Shannon diversity, (d) inverse Simpson diversity, and (e) evenness for each harvest the unaltered (dark points) and sterilized (light points) soil community treatments. (TIF) [file pone.0148015.s006.tif]
